# Analysis of the network structure characteristics and influencing factors of regional tourism economy

**DOI:** 10.1371/journal.pone.0318243

**Published:** 2025-02-18

**Authors:** Xiao-Bing Feng

**Affiliations:** College of Tourism and Geographic Sciences, Leshan Normal University, Leshan, China; Sichuan Agricultural University, CHINA

## Abstract

The tourism economic network is a crucial factor influencing regional tourism interrelations and economic behaviors. This study selects Sichuan Province, China, as the research area and employs social network analysis to evaluate the spatial network structure of its tourism economy from 2015 to 2022. Additionally, the Quadratic Assignment Procedure (QAP) analysis method is used to explain the influencing factors of the tourism economic spatial network structure. The results show that: (1) The volume of regional tourism economic connections is on a rapid growth trajectory, with the radiation capacity and spillover effects of core node areas continuously strengthening. (2) The density of spatial networks in regional tourism economics remains low, showing a trend from loose to tight connections, essentially forming a spatial distribution pattern of “one axis with multiple branches” in tourism economic connections. (3) There are differences in the centrality of tourism economic connection networks among various cities and prefectures within the region, with differing statuses and roles in the tourism economic spatial network. (4) Geographic proximity, tourism resource endowment, economic development levels, and tourism reception capacity positively promote inter-regional tourism economic connections, while the shortest road distance negatively impacts regional tourism economic connections.

## 1. Introduction

Tourism, as a highly interconnected industry, serves as an effective entry point for achieving sustainable regional economic development. The uneven development of regional tourism economies is caused by various factors. Differences in geographical location, tourism resource endowments, and levels of economic development significantly impact regional tourism development [1]. Variations in the structure of the tourism industry lead to different levels of regional tourism economic development [2,3]. The continuous evolution of interregional tourism interactions results in a complex spatial correlation structure of tourism economic development [4], leading to differences in the efficiency of tourism development between regions and significant disparities in interregional tourism economies [5]. As a social and economic phenomenon, tourism activities occur and develop within the material spatial dimension [6]. The spatial structure of tourism is the spatial aggregation state formed by the interaction of tourism objects within a certain geographical area, reflecting the spatial attributes and relationships of tourism activities [7]. Against the backdrop of regional integration development, the gradual formation of regional linkages through the development of tourism resources and the construction of tourism infrastructure has led to increasingly frequent connections within the regional tourism economy, exhibiting a multi-node and complex network structure [8]. The tourism economic network serves as a system that connects source markets and destinations through transportation and information networks. Optimizing the spatial structure of the tourism economic network helps promote comprehensive integration and industrial convergence of tourist destinations [9]. Therefore, analyzing the spatial network structure characteristics of regional tourism economies and their influencing factors can provide references for formulating policies for coordinated regional tourism development, thereby promoting the coordinated development of the regional tourism industry.

Sichuan Province is a significant tourist destination in China. According to the Statistical Bulletin on National Economic and Social Development of Sichuan Province, from 2015 to 2019, tourism revenue in Sichuan increased from 621.05 billion yuan to 1,159.43 billion yuan. However, the characteristic of the tourism industry being large but not strong remains evident, particularly manifested in the imbalances and lack of coordination in tourism economic development. Due to differences in regional economic development levels, tourism resource endowments, and geographical locations, the development of the tourism economy in different areas exhibits distinct characteristics. The development pattern of the tourism industry in different regions, as well as their status and role in the overall tourism economic development, also vary significantly [10]. Chengdu, as the capital city of Sichuan Province, accounts for more than 36% of both tourism revenue and tourist visits in the province, with its level of tourism economic development significantly surpassing that of the other 20 regions. Against the backdrop of the new era of tourism economic development, the coordinated development of regional tourism economies remains a key topic worthy of attention and research. Among the various research perspectives, relational economic geography, with its focus on “relational data” and the economic interactions between regions, has become a new approach in the study of tourism economics.

The rapid development of the tourism industry has led to an implicit connection among various regions in terms of tourist source markets, resource development, and element utilization [11]. Social network analysis quantifies the “relational data” among actors within a network to establish relational models, thereby analyzing the structural relationships and network characteristics among actors in a social network. The tourism industry is a complex system with numerous factors, where these elements are interconnected to form a vast relational network, making it particularly suitable for research through the lens of social network analysis [12]. This method can reveal the internal mechanisms of regional tourism economic development differences, providing a new perspective for studying spatial differences in regional tourism [13]. It has been widely applied in researching the spatial structure, organizational forms, and evolutionary patterns of tourist destinations. Based on this, the article selects Sichuan Province in China as the research area and employs social network analysis to examine the temporal and spatial characteristics of the tourism economic connectivity network structure. Additionally, it evaluates the power status of 21 cities and prefectures in Sichuan within the tourism economic spatial network and explores the influencing factors of the structural evolution of this network, aiming to optimize the spatial structure of the tourism economy in Sichuan and promote coordinated development of the regional tourism economy.

## 2. Literature review

The tourism economy network is an important factor affecting the regional tourism interrelationships and tourism economic behavior [6]. Foreign scholars have been studying the tourism economic network since the 1960s, with research primarily focusing on the characteristics of tourism spatial structures [14–18], the evolution and inherent patterns of tourism network structures [19–23], and the forms of tourism spatial organization [24–28], presenting a research trend that combines theory with practice and qualitative with quantitative aspects. The research subjects encompass a variety of spatial scales, including international, national, provincial, urban, and tourist attractions. Seok et al.[19] utilized social network analysis to explore the dynamic attributes of international tourism spatial frameworks from 2002 to 2014. Xie et al. [29] developed a tourism economic connectivity network among the 28 European Union member states, employing an advanced gravity model in conjunction with social network analysis to assess the intensity of tourism economic linkages from 1995 to 2018, while also examining the spatial structural characteristics of EU tourism economics. Scott et al. [12] performed a comparative analysis of the network structural attributes of four distinct types of tourist destinations in Australia using social network analysis techniques. Park et al. [30] applied trajectory data mining methodologies from mobile big data to investigate the spatial structure of tourism activities across three destinations in South Korea. Cornelissen [31] examined the spatial characteristics of the tourism sector in the Western Cape Province of South Africa. Kelman et al. [15] employed social network analysis to conduct a comprehensive investigation of the network structure and spatial characteristics of tourist flows in the Alpine region. Shih [17] concentrated on 16 self-driving campsites in Nantou, Taiwan, analyzing network structural features such as degree centrality, betweenness centrality, and structural holes concerning tourism destinations. Sugimoto et al. [25] conducted a study on the relationship between tourist flows and urban spatial structure in the Ueno district of Tokyo, Japan, utilizing GPS tracking technology and surveys.

In recent years, with the introduction of methodologies such as social network analysis, there has been a surge in domestic research on the spatial network of tourism economics. The research primarily focuses on the structural characteristics [32–34], evolutionary optimization [35,36], and influencing factors [37,38] of the tourism economic spatial network. Furthermore, the research scope of the tourism economic network mainly involves the China overall area, as well as the more economically developed eastern and central regions of China. The attention to the western regions of China is still relatively lacking. Provincial tourism efficiency networks in China are becoming increasingly complex, with inter-provincial tourism efficiency connections continually on the rise [37]. The tourism economy in China’s provincial regions exhibits a typical overall spatial correlation network structure characterized by low network density and high network connectivity, indicating a significant imbalance in tourism economic development [10]. The total number of inter-provincial tourism economic connections shows a fluctuating upward trend, with economically developed areas such as Beijing, Tianjin, Shanghai, Zhejiang, Guangdong, and Jiangsu demonstrating stronger centrality and greater influence within the network [5]. The tourism flow network density of the Yangtze River Delta urban agglomeration exhibits an overall upward trend, with spatial disparities in tourism network centrality gradually diminishing, and the tourism flow network evolving towards a multi-core structure. Beyond Shanghai, Nanjing, and Hangzhou, new nodes such as Wuxi, Yangzhou, Hefei, and Jiaxing play significant “bridge” and “intermediary” roles within the regional tourism flow network [32]. In contrast, the tourism economic spatial network structure of the urban agglomeration in the middle reaches of the Yangtze River is relatively loose. Wuhan, Changsha, and Nanchang, as the three core cities, maintain more extensive tourism economic connections with other cities, serving as intermediaries and bridges within the tourism economic spatial network structure [33].

In the study of the spatial structure of the tourism economy in the western region of China, the volume of tourism economic connections in Yunnan Province has been increasing year by year, and the spatial network structure of tourism economic connections is tending towards maturity. The Kunming-Dali-Lijiang corridor has developed into the main axis of tourism economic connections. The spatial network of tourism economic connections in Yunnan Province is divided into four cohesive subgroups, with a steady increase in the density of tourism economic connections within and between these subgroups. The connections are becoming increasingly tight, and tourism cooperation and exchanges are becoming more frequent [11]. In Guizhou Province, the structure of tourism economic connections is becoming more rational, with the overall tourism economic network gradually transitioning from loose to tight relationships. The regional tourism development is evolving from a unipolar model centered around Guiyang to a multipolar model, with Zunyi City, Qiandongnan Prefecture, and Anshun City emerging as new growth poles [39]. In Sichuan Province, the overall density of the tourism economic spatial network is relatively low, and the tourism economic connections between regions are not closely knit. There is a notable regional imbalance in tourism economic development, with Chengdu serving as the core node of the tourism economic spatial network, exhibiting the strongest capacity to attract and diffuse tourist flows [40]. The tourism regions in Sichuan display a typical “core-periphery” spatial structure, characterized by a significant development gap between core and peripheral cities. The evolution of the tourism spatial pattern is primarily driven by agglomeration and uneven polarization effects. However, diffusion effects are gradually strengthening, with secondary core cities still in the growth phase [41].

Overall, the existing research on the spatial structure characteristics and evolutionary patterns of the tourism economy network has reached a relatively mature stage. However, during the regional tourism economic development process, how do different regions exert power and influence within the spatial structure of the tourism economy? How is their power status within the tourism economic spatial network formed? What factors influence the formation of power status in the tourism economic spatial network? These are all questions worthy of in-depth discussion. Coordinated regional tourism economic development is an objective requirement for developing Sichuan’s tourism industry and studying regional tourism development within the context of a spatial network is a new direction. Current research on the spatial structure of Sichuan’s tourism economy focuses on data analysis of spatial structure characteristics, lacking analysis of the evolutionary trends and driving factors of these structural characteristics. In terms of research methods, traditional geographical spatial analysis perspectives are predominant, with fewer studies analyzing from a social network perspective based on relational data.

## 3. Research design

### 3.1. Research area

Sichuan Province is located in the southwestern part of China, covering an area of 486,000 square kilometers and administratively divided into 21 cities (prefectures). It is a significant tourist destination in China, boasting three UNESCO World Natural Heritage sites, one UNESCO World Cultural Heritage site, and one site recognized for both cultural and natural heritage. The province is home to 928 national A-level tourist attractions, including 16 five-A-level sites. According to the Statistical Bulletin of National Economic and Social Development of Sichuan Province, the total tourism revenue in 2019 reached 1,159.43 billion yuan, with 750 million domestic tourists and a domestic tourism revenue of 1,145.45 billion yuan. It also welcomed 4.148 million inbound tourists, resulting in foreign exchange earnings from tourism amounting to 2.02 billion US dollars. In 2020, impacted by the COVID-19 pandemic, the province’s total tourism revenue was 717.33 billion yuan, with 450 million domestic tourists, generating domestic tourism revenue of 717.01 billion yuan. The number of inbound tourists was 246,000, with foreign exchange earnings from tourism reaching 46.791 million US dollars.

### 3.2. Research methods

#### 3.2.1. Tourism gravity model.

The tourism gravity model originates from the law of universal gravitation in physics. Zipf introduced this law into the urban spatial dimension to establish the gravity model [42], which has been widely applied in the study of interactions between cities. In the formula: $R_{ij}$ represents the tourism economic connection between spatial units i and j, $P_{i}$ is the number of tourists in spatial unit i, $Q_{i}$ is the tourism revenue in spatial unit i, and $D_{ij}$ is the distance between spatial units i and j*.* $K_{ij}$ is the modified gravitational coefficient to characterize the degree and direction of variation in the attractiveness of the regional tourism economy.


$$R_{ij}=K_{ij}\cdot \frac{\sqrt{P_{i}\times Q_{i}}\sqrt{P_{j}\times Q_{j}}}{D_{ij}^{2}},\;K_{ij}=\frac{Q_{i}}{Q_{i}+Q_{j}}\left(i\neq j\right)$$ (1)

$R_{i}$ represents the tourism economic connectivity of city i, encompassing the total tourism economic interactions between city i and other cities within the region. It reflects the significance and role of city i within the regional tourism economy.


$${\text{R}}_{\text{i}}=\overset{\text{n}}{\underset{\text{j}=1}{\sum }}{\text{R}}_{\text{ij}}$$ (2)

#### 3.2.2. Network density.

Network density is a crucial metric for assessing the close relationships among members within a regional spatial network. A higher network density indicates stronger tourism spatial connections between regions, thereby enhancing the overall competitive capacity of the region. The formula for calculating network density is as follows [43].


$$\text{D}=\frac{\underset{i=1}{\sum^{n}}\underset{j=1}{\sum^{n}}d\left(n_{i},\;n_{j}\right)}{n\left(n-1\right)}$$ (3)

In the formula: D represents the overall density of the network space, and n is the number of nodes in the network space. If there is a connection between node i and node j, then $d\left(n_{i}n_{j}\right)$ = 1, otherwise it is 0. $n\left(n-1\right)$ represents the total number of theoretically existing relationships in the overall network. $\underset{i=1}{\sum^{n}}\underset{j=1}{\sum^{n}}d\left(n_{i},\;n_{j}\right)$ represents the sum of actual relationships present in the spatial network.

#### 3.2.3. Degree centrality.

Degree centrality reflects the ability of individual members within the network to interact with other members. If a member has direct connections with many members in the network, then that member occupies a central position in the network, which means having a higher degree centrality [43]. $C_{i}$ represents the degree centrality of region i, and $R_{\text{ij}}$ is the tourism economic connection between region i and region j.


$$C_{i}=\overset{n}{\underset{j=1}{\sum }}R_{\text{ij}}$$ (4)

#### 3.2.4. Betweenness centrality.

Betweenness centrality measures the extent to which a member in the network can control other members. A higher betweenness centrality indicates greater control over tourism flows from other regions within the tourism spatial network. If a member’s betweenness centrality in the spatial network is 0, it means that the member cannot control any actors [43]. In the formula, $B_{i}$ represents the betweenness centrality of unit i, $D_{jk}$ represents the number of shortest paths between unit j and unit k, and $D_{jk}\left(i\right)$ represents the number of shortest paths between unit j and unit k passing through unit i.


$$B_{i}=\underset{i=j\neq \text{k}}{\sum }\frac{D_{jk}\left(i\right)}{D_{jk}}$$ (5)

#### 3.2.5. Closeness centrality.

If a point has very short distances to all other points in the network, it is said to have a high closeness centrality. Closeness centrality is a measure of an actor’s independence from the control of other actors, indicating an actor’s ability to remain “uncontrolled” by others [43]. The formula for calculating closeness centrality is as follows, where $d_{\text{ij}}$ is the shortest path distance between points i and j (i.e., the number of lines contained in the shortest path).


$${\text{C}}_{APi}^{-1}=\overset{n}{\underset{j=1}{\sum }}d_{\text{ij}}$$ (6)

### 3.3. Data sources

The data on tourism revenue and tourist numbers for the 21 cities and prefectures in Sichuan Province were sourced from the Sichuan Statistical Yearbook for 2016-2021 and 2023. The Sichuan Statistical Yearbook is missing data on tourism revenue and tourist arrivals in Sichuan Province for 2021. The spatial distances between these 21 cities and prefectures are based on the shortest road distances calculated using Gaode Map for driving. The relevant indicators for the factors influencing the spatial network structure of the tourism economy were also derived from the Sichuan Statistical Yearbook. Using the formula (1), we calculated the tourism economic connectivity for the 21 cities and prefectures in Sichuan Province from 2015 to 2020 and 2022, constructing seven 21x21 tourism economic connectivity matrices. To ensure temporal and spatial comparability of the data, the average value of tourism economic linkages was used as a threshold. The tourism economic linkage matrix was dichotomized using the social network analysis software Ucinet, converting the relational matrix from multivalued relational data into binary “0-1” relational data. When the tourism economic linkage between two spatial units exceeds the average value, it is marked as 1, indicating a relationship. Conversely, if it is below the average, it is marked as 0, indicating no connection. The analysis of the spatial structure characteristics of tourism economics in the text will be based on the processed “0-1” relational matrix, utilizing Ucinet software for the analysis.

## 4. Research content

### 4.1. Analysis of the spatial connection of tourism economy

#### 4.1.1. Analysis of the degree of tourism economic connection.

Using the formula (1), the tourism economic connectivity between the 21 cities and prefectures in Sichuan Province was calculated from 2015 to 2022. The details of the tourism economic connections for 2015, 2019, 2020, and 2022 can be found in Tables 1-4. The specific figures in the tables represent the tourism economic connectivity between two cities or prefectures in Sichuan Province. For instance, in the 2015 tourism economic connectivity matrix for Sichuan Province, the connectivity between Chengdu and Zigong was 135.2, while the connectivity from Zigong to Chengdu was 16.4. A higher number indicates a stronger tourism economic connection between the two cities or prefectures. From Table 1, it is evident that in 2015, the tourism economic connections among the 21 cities and prefectures in Sichuan Province were primarily centered around Chengdu, with the strongest connection in the direction of Chengdu-Meishan, achieving a connectivity score of 882.5. Aside from Meishan, Chengdu also maintained a high level of tourism economic connectivity with Ziyang, Deyang, Leshan, and Mianyang, with connectivity scores of 629.8, 494.8, 381.4, and 342.7, respectively. The tourism economic connections between Zigong and Neijiang, Mianyang and Deyang, Neijiang and Zigong, as well as Meishan and Chengdu, were also relatively close. Conversely, the cities and prefectures of Panzhihua, Guangyuan, Bazhong, Dazhou, Aba, Ganzi, and Liangshan, due to their peripheral geographical locations within Sichuan Province and greater distances from the central city, exhibited weaker tourism economic connections with other cities and prefectures.

**Table 1:** Tourism economic connection intensity in Sichuan Province, 2015.

City	Chengdu	Zigong	Panzhihua	Luzhou	Deyang	Mianyang	Guangyuan	Suining	Neijiang	Leshan	Nanchong	Meishan	Yibin	Guang’an	Dazhou	Ya’an	Bazhong	Ziyang	Aba	Ganzi	Liangshan
Chengdu		135.2	8.4	75.0	494.8	342.7	53.1	168.7	132.3	381.4	110.3	882.5	98.5	55.4	16.1	198.3	23.3	629.8	52.9	30.5	28.5
Zigong	16.4		0.5	40.8	4.4	3.9	2.0	11.2	162.2	27.3	6.4	13.8	60.5	5.0	1.4	5.4	1.7	22.3	1.4	1.5	1.4
Panzhihua	0.8	0.4		0.4	0.4	0.4	0.3	0.4	0.4	0.6	0.3	0.6	0.4	0.3	0.1	0.7	0.2	0.5	0.3	0.3	5.6
Luzhou	9.2	41.6	0.5		3.2	3.1	1.8	7.3	36.0	10.7	4.7	6.6	38.4	4.8	1.6	3.4	1.4	11.5	1.2	1.2	1.2
Deyang	37.5	2.7	0.3	2.0		71.8	4.4	6.5	3.7	4.0	4.4	8.0	2.1	2.3	0.9	3.9	1.6	10.1	1.6	1.0	0.8
Mianyang	57.4	5.4	0.7	4.2	158.3		18.4	21.5	6.5	8.5	19.0	11.2	4.5	8.1	2.7	7.0	5.0	15.3	3.7	2.0	2.0
Guangyuan	5.3	1.6	0.3	1.5	5.8	11.0		5.3	2.1	1.8	8.1	2.3	1.4	3.9	3.0	1.6	11.4	2.7	1.1	0.6	0.7
Suining	20.6	11.3	0.5	7.2	10.4	15.7	6.5		18.1	6.4	71.5	8.7	6.4	27.3	4.5	3.6	5.9	21.1	1.8	1.2	1.2
Neijiang	11.0	111.3	0.3	24.2	4.0	3.2	1.7	12.3		10.9	5.7	7.6	17.5	4.5	1.2	3.1	1.4	28.7	0.9	1.0	0.9
Leshan	92.7	54.9	1.6	21.1	13.0	12.4	4.3	12.7	31.8		8.3	52.8	34.9	6.4	2.0	51.8	2.3	45.5	3.5	7.2	5.4
Nanchong	17.3	8.2	0.5	6.0	9.1	17.8	12.8	91.6	10.7	5.3		6.8	5.6	77.0	10.8	3.3	12.0	11.8	2.0	1.1	1.3
Meishan	99.0	12.8	0.7	6.0	11.8	7.5	2.6	8.0	10.2	24.4	4.9		7.6	3.4	1.2	28.3	1.4	35.1	2.5	3.7	2.3
Yibin	15.9	80.9	0.7	50.4	4.4	4.3	2.3	8.5	34.0	23.2	5.8	11.0		4.7	1.5	6.5	1.7	14.0	1.8	1.9	1.9
Guang’an	6.6	4.9	0.3	4.6	3.6	5.8	4.7	26.7	6.4	3.1	58.8	3.7	3.5		9.3	1.9	6.6	6.8	1.1	0.7	0.8
Dazhou	0.9	0.6	0.1	0.7	0.7	0.9	1.7	2.0	0.8	0.5	3.8	0.6	0.5	4.3		0.4	5.4	0.8	0.2	0.2	0.2
Ya’an	14.7	3.3	0.5	2.1	3.8	3.1	1.2	2.2	2.8	15.8	1.6	18.7	3.0	1.2	0.5		0.7	5.8	1.2	6.6	2.2
Bazhong	1.5	0.9	0.1	0.7	1.3	1.9	7.3	3.1	1.1	0.6	4.9	0.8	0.7	3.5	6.3	0.6		1.2	0.3	0.2	0.3
Ziyang	68.0	19.8	0.5	10.1	14.3	9.9	2.9	18.6	37.2	20.2	8.1	33.8	9.4	6.2	1.6	8.4	2.1		2.2	2.0	1.6
Aba	7.4	1.6	0.4	1.4	3.0	3.1	1.5	2.0	1.6	2.0	1.8	3.1	1.5	1.3	0.6	2.2	0.8	2.8		0.9	1.0
Ganzi	1.6	0.7	0.2	0.5	0.7	0.6	0.3	0.5	0.6	1.6	0.4	1.7	0.6	0.3	0.2	4.7	0.2	1.0	0.3		0.9
Liangshan	3.5	1.4	6.9	1.2	1.3	1.4	0.8	1.2	1.3	2.7	1.0	2.5	1.4	0.8	0.4	3.6	0.5	1.8	0.9	2.0	

From Table 2, it can be seen that between 2015 and 2019, the degree of tourism economic connectivity among the 21 cities and prefectures in Sichuan Province has significantly increased, indicating a closer integration of regional tourism economies. The strength of the tourism economic connection between Chengdu and Meishan, Deyang, Leshan, Mianyang, and Ziyang all exceeds 1000, while the connection with Ya’an, Suining, Neijiang, and Nanchong is also above 400. The tourism economic connections between Zigong and Neijiang, Yibin, Luzhou and Zigong, Neijiang, Yibin, Deyang and Chengdu, Mianyang, Mianyang and Chengdu, Deyang, Suining and Nanchong, Neijiang and Zigong, Leshan and Chengdu, Zigong, Neijiang, Meishan, Yibin, Ya’an, Nanchong and Suining, Guang’an, Meishan and Chengdu, Ya’an, Yibin and Zigong, Luzhou, Neijiang, Leshan, and Guang’an and Nanchong are also relatively close, with connection strengths all greater than 100. From Tables 3-4, it is evident that from 2020 to 2022, the intensity of tourism economic connections among the 21 cities and prefectures in Sichuan Province exhibits a clear downward trend. The number of cities and prefectures with strong tourism economic connections also decreases significantly, with the overall average value of tourism economic connections declining from 786.42 to 443.39. In 2020, the intensity of tourism economic connections between Chengdu and Meishan remains the highest at 1579.6. Except for weak tourism economic connections with Panzhihua, Guang’an, Dazhou, Bazhong, Aba, and Liangshan, Chengdu’s tourism economic ties with the other 14 cities and prefectures have an intensity greater than 100. In 2022, Chengdu’s tourism economic ties with Ziyang and Meishan are the strongest, with intensities of 796.4 and 710.0, respectively.

**Table 2:** Tourism economic connection intensity in Sichuan Province, 2019.

City	Chengdu	Zigong	Panzhihua	Luzhou	Deyang	Mianyang	Guangyuan	Suijning	Neijiang	Leshan	Nanchong	Meishan	Yibin	Guang’an	Dazhou	Ya’an	Bazhong	Ziyang	Aba	Ganzi	Liangshan
Chengdu		446.4	29.8	273.5	2228.8	1195.2	200.6	634.0	466.3	1369.9	428.3	3061.0	421.7	165.9	80.0	823.1	92.4	1111.6	92.8	176.1	87.0
Zigong	41.9		1.6	123.5	15.4	12.3	6.7	35.8	506.8	83.2	20.3	42.4	205.4	13.7	5.7	18.5	5.8	45.1	3.1	6.8	3.7
Panzhihua	2.6	1.5		1.3	1.6	1.6	1.1	1.4	1.4	2.4	1.3	2.3	1.7	0.9	0.7	2.9	0.7	1.2	0.8	1.8	17.7
Luzhou	34.8	167.3	1.9		14.9	13.1	7.9	30.7	143.9	44.8	20.4	26.2	176.8	17.1	8.4	15.1	6.3	28.9	3.3	6.6	4.2
Deyang	218.2	16.0	1.7	11.4		448.7	27.5	39.1	20.8	25.3	27.7	45.8	13.8	11.8	6.4	24.0	9.4	37.7	6.9	8.0	4.2
Mianyang	163.2	17.9	2.4	14.0	625.9		67.4	75.4	22.1	28.5	67.5	37.6	17.0	24.0	12.0	26.6	18.7	31.7	8.3	9.9	5.7
Guangyuan	21.2	6.9	1.2	6.1	27.4	48.9		23.4	8.5	7.8	36.6	9.8	6.7	14.7	15.9	7.2	51.6	7.1	3.2	3.6	2.5
Suining	76.2	45.8	1.9	29.0	48.0	66.4	28.5		73.0	26.6	307.3	34.9	29.3	97.9	23.2	16.3	25.8	53.4	4.9	6.8	4.2
Neijiang	36.1	417.3	1.2	87.4	16.4	12.5	6.8	46.9		40.8	21.8	27.7	71.4	14.7	5.5	12.5	5.8	71.7	2.6	4.9	2.7
Leshan	304.5	196.9	5.9	78.1	57.4	46.3	17.2	49.3	117.1		32.6	192.2	148.6	20.5	10.1	217.4	9.4	95.5	7.7	40.6	17.1
Nanchong	67.9	34.3	2.2	25.4	44.9	78.3	58.1	405.1	44.8	23.2		28.4	27.4	286.4	59.4	15.6	55.1	29.9	5.4	7.1	4.9
Meishan	311.2	45.9	2.6	20.9	47.6	28.0	10.2	29.5	36.5	87.9	18.2		30.3	10.9	5.5	110.9	5.6	80.8	6.4	18.6	7.2
Yibin	74.4	385.6	3.4	245.0	24.8	21.9	11.8	43.0	162.8	117.9	30.5	52.5		20.0	9.1	35.1	9.0	40.0	5.4	13.6	8.1
Guang’an	16.4	14.4	1.0	13.3	12.0	17.4	14.9	80.5	18.8	9.1	178.6	10.5	11.2		35.2	6.1	21.3	12.9	2.3	3.1	2.1
Dazhou	5.6	4.2	0.5	4.6	4.6	6.2	11.6	13.5	5.0	3.2	26.3	3.8	3.6	25.0		2.5	36.5	3.7	1.2	1.4	1.0
Ya’an	68.9	16.5	2.7	9.9	20.5	16.3	6.3	11.3	13.5	81.8	8.2	91.3	16.6	5.2	3.0		3.4	18.9	4.3	43.0	9.4
Bazhong	6.0	4.0	0.5	3.2	6.3	8.9	35.1	14.0	4.9	2.8	22.7	3.6	3.3	14.1	34.0	2.6		3.9	1.2	1.4	1.0
Ziyang	52.2	22.6	0.6	10.7	18.1	10.9	3.5	20.9	43.6	20.2	8.9	37.3	10.7	6.1	2.5	10.6	2.8		2.0	3.3	1.5
Aba	4.5	1.6	0.4	1.3	3.4	2.9	1.6	2.0	1.6	1.7	1.6	3.0	1.5	1.1	0.8	2.5	0.9	2.0		1.3	0.8
Ganzi	13.8	5.7	1.6	4.1	6.4	5.7	3.0	4.4	5.0	14.3	3.5	14.4	6.0	2.4	1.6	40.3	1.7	5.5	2.1		6.3
Liangshan	9.8	4.4	22.5	3.7	4.9	4.7	2.9	3.9	4.0	8.7	3.5	8.0	5.2	2.4	1.7	12.7	1.7	3.6	1.9	9.1	

**Table 3:** Tourism economic connection intensity in Sichuan Province, 2020.

City	Chengdu	Zigong	Panzhihua	Luzhou	Deyang	Mianyang	Guangyuan	Suijning	Neijiang	Leshan	Nanchong	Meishan	Yibin	Guang’an	Dazhou	Ya’an	Bazhong	Ziyang	Aba	Ganzi	Liangshan
Chengdu		172.1	14.9	130.4	996.1	765.6	119.8	384.3	264.0	853.3	294.4	1579.6	228.9	69.8	48.9	476.6	52.9	516.0	74.3	108.1	33.8
Zigong	13.5		0.5	41.2	5.3	4.5	2.7	13.4	187.9	28.9	7.7	14.8	70.6	4.4	2.2	6.9	2.2	15.8	1.4	2.6	1.2
Panzhihua	1.6	0.7		0.7	0.9	1.0	0.7	0.9	0.8	1.4	0.8	1.3	1.0	0.5	0.4	1.7	0.4	0.6	0.5	1.1	8.9
Luzhou	18.1	72.7	0.9		7.3	7.5	4.7	17.6	79.1	24.8	12.3	13.6	94.0	7.8	4.9	8.4	3.5	14.0	2.3	3.9	1.9
Deyang	89.4	6.1	0.7	4.7		206.7	13.9	18.3	9.5	11.1	13.2	19.8	5.9	4.6	3.1	11.1	4.4	16.0	3.9	3.9	1.6
Mianyang	163.0	12.3	2.0	11.5	490.6		66.2	73.4	19.8	28.6	71.8	32.1	15.7	17.6	11.4	24.3	16.8	23.6	9.4	9.4	4.1
Guangyuan	18.4	4.3	0.9	4.5	19.3	42.5		19.9	6.7	6.8	33.6	7.4	5.4	9.7	13.2	5.8	40.8	4.8	3.1	3.1	1.6
Suining	64.1	28.5	1.4	21.2	34.0	57.5	24.9		58.0	22.9	281.2	26.5	23.6	64.7	19.4	13.2	20.6	36.1	4.9	5.7	2.7
Neijiang	27.3	247.8	0.8	59.0	11.0	9.6	5.5	35.9		30.9	17.6	19.3	51.9	9.2	4.2	9.1	4.2	45.3	2.2	3.8	1.7
Leshan	295.4	127.4	4.7	62.1	42.7	46.5	16.4	47.5	103.5		35.0	160.1	134.3	14.3	9.5	196.8	8.4	69.3	9.0	38.6	11.4
Nanchong	76.5	25.5	2.0	23.1	38.3	87.6	62.6	438.3	44.2	26.3		26.7	28.1	229.2	62.1	15.8	54.5	24.3	6.8	7.5	3.8
Meishan	190.9	22.9	1.5	11.9	26.7	18.2	7.0	19.2	22.5	55.9	12.4		18.5	5.7	3.6	69.6	3.5	44.1	4.9	12.2	3.7
Yibin	51.0	200.7	2.1	151.3	14.7	16.4	8.8	31.6	112.0	86.5	24.1	34.1		11.1	6.7	24.6	6.3	23.3	4.8	10.0	4.3
Guang’an	6.5	5.2	0.4	5.3	4.8	7.7	7.2	36.3	8.3	3.9	82.4	4.4	4.7		16.3	2.7	9.6	5.2	1.2	1.4	0.8
Dazhou	4.9	2.8	0.4	3.5	3.4	5.4	10.5	11.7	4.0	2.7	24.1	3.0	3.0	17.6		2.1	29.7	2.6	1.2	1.2	0.7
Ya’an	54.3	10.0	1.9	6.9	14.1	13.0	5.2	9.0	10.1	64.7	6.9	65.6	12.6	3.3	2.4		2.5	12.3	3.9	33.9	5.9
Bazhong	4.6	2.5	0.4	2.2	4.3	6.9	28.6	10.8	3.5	2.1	18.3	2.5	2.5	9.0	25.7	2.0		2.5	1.0	1.1	0.6
Ziyang	25.0	9.8	0.3	4.9	8.6	5.4	2.0	10.5	21.2	9.7	4.5	17.7	5.1	2.7	1.3	5.2	1.4		1.1	1.7	0.7
Aba	7.4	1.8	0.5	1.7	4.3	4.4	2.5	2.9	2.2	2.6	2.6	4.0	2.2	1.3	1.1	3.4	1.2	2.3		1.8	1.0
Ganzi	12.1	3.8	1.2	3.1	4.8	5.0	2.7	3.9	4.1	12.5	3.2	11.3	5.0	1.7	1.3	33.3	1.4	3.9	2.0		4.4
Liangshan	3.1	1.4	7.8	1.2	1.7	1.7	1.2	1.5	1.5	3.0	1.3	2.8	1.8	0.8	0.7	4.8	0.7	1.3	0.9	3.6	

**Table 4:** Tourism economic connection intensity in Sichuan Province, 2022.

City	Chengdu	Zigong	Panzhihua	Luzhou	Deyang	Mianyang	Guangyuan	Suijning	Neijiang	Leshan	Nanchong	Meishan	Yibin	Guang’an	Dazhou	Ya’an	Bazhong	Ziyang	Aba	Ganzi	Liangshan
Chengdu		67.9	5.1	85.8	423.4	565.8	34.6	107.9	208.1	279.8	100.8	710.0	162.6	37.7	17.9	220.7	14.2	796.4	17.4	29.8	4.4
Zigong	4.8		0.2	15.6	1.8	1.6	0.8	3.7	78.3	10.0	2.2	5.7	27.2	1.7	0.6	2.2	0.6	11.4	0.3	0.7	0.2
Panzhihua	0.3	0.2		0.2	0.2	0.2	0.1	0.2	0.2	0.3	0.1	0.3	0.2	0.1	0.1	0.4	0.1	0.3	0.1	0.2	1.0
Luzhou	19.1	49.6	0.6		5.4	8.2	2.6	10.4	91.1	20.0	8.7	11.5	105.7	7.1	3.0	6.6	1.8	28.3	1.0	2.1	0.5
Deyang	36.2	2.1	0.3	2.1		86.2	4.2	5.6	4.6	4.3	4.3	8.5	2.6	2.0	0.9	4.0	1.3	13.5	0.9	1.2	0.3
Mianyang	253.5	10.4	1.6	16.4	452.5		43.8	51.8	31.7	27.9	65.1	33.3	25.3	20.4	9.0	24.7	10.1	67.4	5.0	6.1	1.2
Guangyuan	4.2	0.9	0.2	1.2	4.4	11.3		3.5	2.1	1.5	6.4	1.9	1.5	2.6	2.4	1.3	7.0	2.8	0.4	0.5	0.2
Suining	10.7	5.3	0.2	4.6	6.5	11.5	3.8		14.6	3.9	41.5	5.6	5.0	14.5	2.9	2.4	3.1	17.5	0.6	0.9	0.2
Neijiang	41.9	224.7	0.7	82.2	10.8	14.3	4.1	29.5		35.7	17.6	22.2	80.4	11.0	3.4	9.7	2.9	115.6	1.3	2.8	0.6
Leshan	52.4	26.6	0.9	16.8	9.4	11.7	2.7	7.3	33.3		5.7	37.7	34.8	3.8	1.7	43.0	1.3	44.8	1.2	6.1	0.9
Nanchong	21.2	6.6	0.5	8.3	10.6	30.6	12.8	88.1	18.5	6.4		8.1	10.1	76.7	13.9	4.4	10.6	19.6	1.1	1.5	0.4
Meishan	66.8	7.7	0.5	4.9	9.4	7.0	2.0	5.3	10.4	19.0	3.6		7.5	2.3	1.0	23.9	1.0	37.1	1.1	3.4	0.6
Yibin	62.5	149.4	1.5	182.4	11.9	21.8	5.1	19.3	153.6	71.4	18.5	30.8		11.2	4.6	21.6	3.4	57.9	2.2	5.7	1.2
Guang’an	4.1	2.6	0.2	3.4	2.5	4.9	3.0	15.8	5.9	2.2	39.4	2.7	3.1		6.8	1.4	3.8	6.5	0.4	0.6	0.2
Dazhou	2.1	1.0	0.1	1.6	1.2	2.4	3.0	3.5	2.0	1.1	7.9	1.2	1.4	7.5		0.8	8.0	2.3	0.3	0.3	0.1
Ya’an	29.6	4.2	0.8	4.0	6.4	7.4	1.8	3.3	6.4	30.8	2.8	34.0	7.5	1.8	0.9		0.9	13.8	1.1	11.9	1.1
Bazhong	0.6	0.4	0.1	0.4	0.7	1.0	3.8	1.4	0.6	0.3	2.2	0.4	0.4	1.6	2.9	0.3		0.8	0.1	0.1	0.1
Ziyang	115.7	23.6	0.7	18.4	22.9	21.9	4.2	25.6	83.4	34.8	13.5	57.3	21.9	8.7	2.8	15.0	2.7		1.8	3.6	0.7
Aba	1.2	0.3	0.1	0.3	0.7	0.7	0.3	0.4	0.4	0.4	0.4	0.8	0.4	0.3	0.1	0.5	0.2	0.8		0.2	0.1
Ganzi	1.8	0.6	0.2	0.6	0.8	0.8	0.4	0.5	0.8	2.0	0.4	2.2	0.9	0.3	0.2	5.3	0.2	1.5	0.2		0.4
Liangshan	0.1	0.1	0.4	0.0	0.1	0.1	0.0	0.0	0.1	0.1	0.0	0.1	0.1	0.0	0.0	0.2	0.0	0.1	0.0	0.1	

#### 4.1.2. Analysis of tourism economic connectivity.

Using the formula (2), the tourism economic connectivity of 21 cities and prefectures in Sichuan Province from 2015 to 2020 and 2022 is calculated. From Table 5, it can be seen that the regional tourism economic connectivity in Sichuan Province showed a rapid growth trend from 2015 to 2019. In 2015, the overall mean of tourism economic connectivity was 362.99, which increased to 1283.69 in 2019, a growth rate of 254%. However, in 2020, due to the impact of the COVID-19 pandemic, the desire and behavior of tourists were restricted, leading to a significant overall decline in tourism economic connectivity in Sichuan Province. The overall mean of tourism economic connectivity in 2020 was only 786.42, which was lower than the tourism economic connectivity in 2018. In 2015, Chengdu accounted for 51.39% of the tourism economic connectivity, far exceeding other cities. Leshan and Zigong, ranked second and third in terms of tourism economic connectivity, accounted for only 6.09% and 5.11% respectively. Yibin, Nanchong, Mianyang, Suining, Meishan, Neijiang, and Ziyang also had a tourism economic connectivity of over 3%.

**Table 5:** Tourism economic connectivity in Sichuan Province from 2015 to 2022.

City	2015		2019		2020		2022	
	Connections	PCT	Connections	PCT	Connections	PCT	Connections	PCT
Chengdu	3917.37	51.39%	13384.39	49.65%	7183.93	43.50%	3890.43	41.78%
Leshan	464.44	6.09%	1664.43	6.17%	1432.79	8.68%	341.79	3.67%
Yibin	275.44	3.61%	1313.80	4.87%	824.38	4.99%	835.98	8.98%
Nanchong	311.24	4.08%	1303.65	4.84%	1283.36	7.77%	349.84	3.76%
Mianyang	361.31	4.74%	1275.94	4.73%	1103.65	6.68%	1157.27	12.43%
Zigong	389.34	5.11%	1197.67	4.44%	427.79	2.59%	169.47	1.82%
Deyang	169.65	2.23%	1004.60	3.73%	447.99	2.71%	185.02	1.99%
Suining	249.95	3.28%	999.21	3.71%	811.19	4.91%	155.14	1.67%
Meishan	273.63	3.59%	914.87	3.39%	554.77	3.36%	214.38	2.30%
Neijiang	251.31	3.30%	906.90	3.36%	596.26	3.61%	711.47	7.64%
Luzhou	189.36	2.48%	772.42	2.87%	399.48	2.42%	383.23	4.12%
Guang’an	160.16	2.10%	481.25	1.79%	214.49	1.30%	109.54	1.18%
Ya’an	90.85	1.19%	451.10	1.67%	338.35	2.05%	170.56	1.83%
Guangyuan	71.56	0.94%	310.35	1.15%	251.83	1.52%	56.45	0.61%
Ziyang	276.88	3.63%	288.97	1.07%	138.66	0.84%	479.13	5.15%
Bazhong	37.35	0.49%	173.45	0.64%	130.97	0.79%	18.07	0.19%
Dazhou	25.08	0.33%	164.02	0.61%	134.76	0.82%	47.88	0.51%
Ganzi	17.55	0.23%	147.64	0.55%	120.41	0.73%	20.22	0.22%
Liangshan	36.81	0.48%	119.33	0.44%	42.50	0.26%	1.74	0.02%
Panzhihua	13.51	0.18%	46.71	0.17%	25.98	0.16%	4.88	0.05%
Aba	39.95	0.52%	36.83	0.14%	138.66	0.84%	8.68	0.09%
Mean	362.99	4.76%	1283.69	4.76%	786.42	4.76%	443.39	4.76%

Between 2015 and 2019, the proportion of tourism economic connections in Chengdu slightly decreased from 51.39% to 49.65%, yet it maintained a clear core advantage within the region. The proportions of tourism economic connections in Leshan, Mianyang, Panzhihua, and Neijiang showed minimal changes. In contrast, Ziyang, Zigong, Meishan, Guang’an, Liangshan, and Aba exhibited a downward trend in their tourism economic connections, with Ziyang experiencing the most significant decline, dropping from 3.63% to 1.07%. Other cities and prefectures saw a certain increase in their tourism economic connections, with Yibin and Deyang showing the most substantial growth. In 2020, the overall trend of regional tourism economic connections was declining, with a slight improvement in the spatial concentration distribution characteristics. The cities and prefectures with tourism economic connections above the overall average were Chengdu, Leshan, Nanchong, Mianyang, Yibin, and Suining. By 2022, the trend of declining tourism economic connections in Sichuan Province persisted; however, Mianyang, Yibin, Neijiang, Ziyang, and Luzhou showed a noticeable increase in their proportions, while Leshan, Nanchong, and Suining experienced a more pronounced decline.

Overall, the spatial distribution of tourism economic connections in Sichuan Province is relatively concentrated, with Chengdu and other cities accounting for approximately 47% of the province’s total tourism economic connections. Mianyang and Leshan rank second and third in terms of tourism economic connections, but their share with other cities is only about 6%, indicating a significant gap compared to Chengdu. Yibin and Nanchong have tourism economic connection shares above the regional average, around 5%, while Neijiang’s share exceeds 4%. These five cities form the second tier in terms of tourism economic connections in Sichuan Province. The cities of Deyang, Ziyang, Suining, Meishan, Zigong, and Luzhou have tourism economic connection shares between 2% and 4%, while Guang’an, Ya’an, and Guangyuan have shares between 1% and 2%. The cities of Bazhong, Dazhou, Ganzi Prefecture, Liangshan Prefecture, Panzhihua, and Aba Prefecture all have tourism economic connection shares below 1%, indicating that these areas have less interaction with other cities in the region and limited spatial exchange of tourist flows.

### 4.2. Analysis of centrality of tourism economic spatial network

#### 4.2.1. Analysis of density of tourism economic spatial network.

In Table 6, it is evident that in 2015, the spatial network density of Sichuan Province’s tourism economy was 0.162, with an average inter-member distance of 2.091 and a network cohesion index of 0.347. Both network density and cohesion index values range from 0 to 1, with higher values signifying stronger cohesion among network participants. From 2015 to 2019, the spatial network density of Sichuan Province’s tourism economy increased from 0.162 to 0.407, while the average distance between network members decreased from 2.091 to 1.607, and the cohesion index rose from 0.347 to 0.622. This indicates a trend of the tourism economic connections in Sichuan Province evolving from loose to tight. However, in 2020, the spatial network density of the tourism economy in Sichuan Province dropped to 0.314, marking a notable decline from 2019. The average distance between regional members increased from 1.607 to 1.728, and the cohesion index fell from 0.622 to 0.549. This suggests that the COVID-19 pandemic adversely affected the overall integration of the tourism economic spatial network in Sichuan Province, resulting in more fragmented economic connections among regional members. By 2022, both the spatial network density and cohesion index further decreased to 0.210 and 0.346, respectively, indicating a reduction in the spatial interaction of tourist flows among the 21 cities and prefectures in Sichuan Province.

**Table 6:** Overall density of tourism economic spatial network in Sichuan Province.

	Network Density	Average Distance	Network Cohesion
2015	0.162	2.091	0.347
2016	0.186	1.827	0.397
2017	0.255	1.793	0.474
2018	0.329	1.669	0.534
2019	0.407	1.607	0.622
2020	0.314	1.728	0.549
2022	0.210	1.686	0.346

#### 4.2.2. Analysis of centrality of tourism economic spatial network.

Using the social network analysis method, the centrality of the tourism economic spatial network in Sichuan Province is analyzed from the three indicators of degree centrality(DC), betweenness centrality(BC), and closeness centrality(CC), and the calculation results are shown in Table 7.

**Table 7:** Analysis of centrality of tourism economic spatial network in Sichuan Province.

City	2015			2019			2020			2022		
	DC	CC	BC	DC	CC	BC	DC	CC	BC	DC	CC	BC
Chengdu	90	33.333	58.684	100	100	36.654	95	50	32.918	75	16.667	17.982
Zigong	30	27.778	0.263	50	66.667	0.285	40	39.216	0.075	30	15.504	0
Panzhihua	0	0	0	10	52.632	0	0	0	0	0	0	0
Luzhou	25	27.397	0	45	64.516	0	35	38.462	0	30	15.504	0
Deyang	10	26.316	0	55	68.966	0.899	35	38.462	0.211	15	15.152	0
Mianyang	25	27.397	1.316	65	74.074	2.309	50	40.816	1.465	60	16.260	6.684
Guangyuan	10	26.316	0	30	58.824	0.105	30	37.736	0.263	10	15.038	0
Suining	30	27.778	1.754	75	80	4.176	75	45.455	7.321	30	15.504	0
Neijiang	35	28.169	1.14	60	71.429	0.65	50	40.816	0.497	50	16.000	1.368
Leshan	40	28.571	2.368	70	76.923	2.689	65	43.478	4.242	45	15.873	0.982
Nanchong	20	27.027	0.175	75	80	4.176	75	45.455	7.321	35	15.625	0.702
Meishan	20	27.027	0.175	65	74.074	1.893	55	41.667	1.299	35	15.625	0.211
Yibin	25	27.397	0	65	74.074	1.066	50	40.816	0.966	55	16.129	2.281
Guang’an	15	26.667	0	45	64.516	0.816	15	35.714	0	15	15.152	0
Dazhou	0	0	0	25	57.143	0	20	36.364	0	0	0.000	0
Ya’an	15	26.667	0	45	64.516	0.775	30	37.736	0.592	25	15.385	0
Bazhong	5	25.974	0	35	60.606	0.342	25	37.037	0.132	0	0	0
Ziyang	30	27.778	0.965	60	71.429	0.534	40	39.216	0.066	55	16.129	2.947
Aba	5	25.974	0	5	51.282	0	5	34.483	0	0	0	0
Ganzi	5	25.974	0	20	55.556	0	15	35.714	0	5	14.925	0
Liangshan	5	25.974	0	10	52.632	0	5	34.483	0	0	0	0

##### (1) Analysis of degree centrality

As can be seen from Table 7, in the period of 2015-2019, the average degree centrality of the tourism economic spatial network in Sichuan Province increased from 20.952 to 48.095. This indicates a gradual increase in the number of tourism economic connections among the 21 cities and prefectures in Sichuan, reflecting a tightening of tourism economic ties between regions. In 2015, Chengdu exhibited the highest degree centrality, reaching 90, signifying its absolute dominance within the tourism economic spatial network and its closest connections with other nodes. Chengdu demonstrates the strongest spatial aggregation of tourist flows and exerts the most significant tourism radiation effect on surrounding cities. Following Chengdu are Leshan, Neijiang, Zigong, Suining, and Ziyang, indicating that these cities also exhibit considerable polarization and diffusion effects on tourism flows in their surrounding areas. The average centrality value of the tourism economic spatial network is 20.952, and the cities with centrality values above the average are Luzhou, Mianyang, and Yibin; the standard deviation of centrality value is 19.311, indicating that there is a large difference in centrality value among different cities and prefectures. Bazhong, Aba Prefecture, Ganzi Prefecture, Liangshan Prefecture, Panzhihua, and Dazhou have relatively lower centrality values, and their influence on the tourism economy of other cities and prefectures is weaker.

In 2019, Chengdu still had the highest centrality value, reaching 100. This indicates that Chengdu consistently occupies a central position within the regional tourism economic spatial network, playing a pivotal role in the network’s dynamics. The power of Nanchong and Suining in the tourism economic spatial network has significantly increased, while the ranking of Leshan has declined. Meishan, Yibin, Mianyang, Neijiang, and Ziyang are also in a leading position in the regional tourism economic spatial network. In 2020, compared to 2019, Chengdu’s centrality value has decreased, but it still ranks the highest among the 21 cities and prefectures in the region. Nanchong, Suining, and Leshan’s centrality values remain in the second to fourth positions. The centrality values of Meishan, Mianyang, Neijiang, and Yibin are also in leading positions in the region, indicating that these cities and prefectures hold relatively important positions in the tourism economic spatial network of Sichuan Province. The standard deviation of centrality value is 24.647, which has increased significantly compared to 2015 and 2019, indicating that the difference in centrality value among the 21 cities and prefectures in Sichuan Province is increasing.

Compared to 2020, the degree centrality of Ziyang, Mianyang, and Yibin increased in 2022, indicating that these three cities are gaining importance within the spatial network of the tourism economy in Sichuan Province. The degree centrality of Neijiang, Guang’an, and Panzhihua remained unchanged, while the other 15 cities and prefectures experienced a noticeable decline. Chengdu continues to have the highest degree centrality, reaching 75, followed by Mianyang, Yibin, Ziyang, Neijiang, and Leshan, which hold relatively important positions in the spatial network of Sichuan’s tourism economy. The average degree centrality is 27.143, with regions such as Nanchong, Meishan, Zigong, Luzhou, and Suining also above this average. The degree centrality values for Panzhihua, Dazhou, Bazhong, Aba Prefecture, and Liangshan Prefecture are 0, placing them at the bottom of the regional ranking. The standard deviation of degree centrality is 22.550, showing a slight decrease compared to 2020, which suggests that the differences in degree centrality among the 21 cities and prefectures in Sichuan Province are narrowing.

##### (2) Analysis of betweenness centrality

As can be seen from Table 7, in terms of the spatial network of the tourism economy in Sichuan Province in 2015, Chengdu has the highest betweenness centrality, reaching 58.684, far exceeding the other 20 cities and prefectures. It shows that Chengdu plays the largest role in acting as a bridge in the spatial network of the tourism economy and has the strongest control over other regions. It plays an intermediary role in the spatial movement of tourist flows in Sichuan Province. The overall mean value of betweenness centrality is 3.183, and the betweenness centrality of other cities except Chengdu is lower than the mean value. It indicates that these cities have weak control over the transfer of tourist flows and do not play an intermediary role in the spatial network of the tourism economy. Leshan has a betweenness centrality of 2.368, ranking second, followed by Suining, Mianyang, Neijiang, Ziyang, Zigong, Nanchong, and Meishan. From 2015 to 2019, the mean and standard deviation of betweenness centrality in the spatial network of the tourism economy in Sichuan Province showed a decreasing trend, indicating that the differences in the betweenness centrality of the 21 cities and prefectures are narrowing.

Chengdu exhibited the highest betweenness centrality among the cities and prefectures in 2019, although its value declined from 58.684 to 36.654. Other cities and prefectures, such as Neijiang and Ziyang, also experienced a reduction in betweenness centrality, while 12 cities and prefectures saw an increase. This trend indicates that a growing number of cities and prefectures are positioned along the routes of tourism economic linkages with other regions, serving intermediary functions to varying degrees. Cities and prefectures with a betweenness centrality of zero included Panzhihua, Luzhou, Dazhou, Aba, Ganzi, and Liangshan. In 2020, the average betweenness centrality within Sichuan Province’s tourism economic spatial network was 2.732, with Chengdu, Suining, Nanchong, and Leshan surpassing the regional average. By 2022, the average betweenness centrality had decreased to 1.579, with Chengdu, Mianyang, Ziyang, and Yibin exceeding the regional average. This suggests that these cities and prefectures play pivotal roles in the spatial redistribution of tourist flows within Sichuan Province. The lower betweenness centrality values of other cities and prefectures reflect their limited capacity to influence tourist flows from other regions. The standard deviation of betweenness centrality was 3.982, marking a significant decrease compared to 2020, which implies that the disparities in betweenness centrality among Sichuan’s 21 cities and prefectures continue to diminish.

##### (3) Analysis of closeness centrality

From 2015 to 2019, the closeness centrality mean of the tourism economic spatial network in Sichuan Province increased from 27.343 to 67.612. This indicates that the tourism economic connections between regions in Sichuan have become increasingly fluid, allowing more areas to freely engage in tourism economic interactions. As can be seen from Table 7, Chengdu exhibited the highest closeness centrality in 2015, suggesting that it maintained the shortest distance in tourism economic connections with other regions, thereby facilitating superior liquidity in tourism economics and rendering it less susceptible to control by other cities and prefectures. Except for Panzhihua and Dazhou, whose closeness centrality was 0, the closeness centrality of other cities and states was relatively close, ranging from 25 to 29. In 2019, Chengdu continues to have the highest closeness centrality at 100, playing an absolutely central actor role in the spatial network of the tourism economy. Suining and Nanchong both had a closeness centrality of 80. This indicates that the tourism economic connections between these three cities and other regions within the tourism economic spatial network are relatively convenient, with tourist flows being less constrained by other cities. In addition, cities and states with a closeness centrality above the overall mean include Leshan, Mianyang, Meishan, Yibin, Neijiang, Ziyang, and Deyang, where the spatial movement of tourists is relatively more unrestricted.

The differences in closeness centrality among the 21 nodes in Sichuan Province’s tourism economic spatial network are relatively small, compared to degree centrality and betweenness centrality. After 2019, both the overall mean and standard deviation of closeness centrality in Sichuan’s tourism economic spatial network showed a significant downward trend, indicating a noticeable reduction in the differences in closeness centrality among cities and prefectures. The imbalance in regional tourism economic development is diminishing, and the degree of tourism integration is improving. In 2020, Chengdu had the highest closeness centrality, reaching 50. Suining, Nanchong, Leshan, Meishan, Mianyang, and Yibin ranked 2nd to 7th in the region, all above the regional average, suggesting that these cities and prefectures are less constrained by others in the tourism economic spatial network. Panzhihua’s closeness centrality value was 0, indicating that its tourism economic connections with other cities and prefectures are not smooth, making it highly susceptible to control by other regions. In 2022, Chengdu, Mianyang, Yibin, Ziyang, and Neijiang ranked 1st to 5th in the region in terms of closeness centrality, with values of 16.667, 16.26, 16.129, 16.129, and 16, respectively. Except for Panzhihua, Dazhou, Bazhong, Aba Prefecture, and Liangshan Prefecture, which had a closeness centrality of 0, the differences in closeness centrality among other cities and prefectures were not significant.

### 4.3. Evaluation of power status of tourism economic spatial network

The power status is primarily used to evaluate and measure the influence of a city or prefecture within the overall tourism economic spatial network. This is mainly assessed through degree centrality, betweenness centrality, and closeness centrality, with the specific calculation formula as follows. Where Ni represents the power status of the city or prefecture i in the tourism economic spatial network. A larger $N_{i}$ value indicates a higher power status and greater influence of city or prefecture i within the tourism economic spatial network. $DC_{i}$, $BC_{i}$, and $CC_{i}$ respectively represent the degree centrality, betweenness centrality, and closeness centrality of city or prefecture i, while a, b, and c represent the weights of degree centrality, betweenness centrality, and closeness centrality, respectively. It is generally considered that in the power status of the tourism economic spatial network, the three are equally important, and thus their weights are all assigned a value of 1/3.


$$N_{i}=aDC_{i}+bBC_{i}+cCC_{i}$$ (7)

To eliminate the dimensional differences among indicators and the impact of data size and scale, the values of degree centrality, betweenness centrality, and closeness centrality were standardized before calculating the power status of the tourism economic spatial network. Using the formula (7), the power status evaluation values for the 21 cities and prefectures in Sichuan Province’s tourism economic spatial network from 2015 to 2022 were obtained. By employing the K-means clustering analysis method and setting the number of clusters to 5, the power status of the 21 cities and prefectures in Sichuan Province as tourism destinations was classified as follows: core tourism destinations, sub-core tourism destinations, important tourism destinations, general tourism destinations, and edge tourism destinations. For details, refer to Table 8.

**Table 8:** Evaluation of power status of tourism economic spatial network in Sichuan Province.

City	Power Status (2015)	Power Status (2019)	Power Status (2020)	Power Status (2022)
Chengdu	Core Destination	Core Destination	Core Destination	Core Destination
Neijiang	Sub-core Destination	Important Destination	Important Destination	Sub-core Destination
Mianyang	Important Destination	Important Destination	Important Destination	Sub-core Destination
Yibin	Important Destination	Important Destination	Important Destination	Sub-core Destination
Ziyang	Important Destination	Important Destination	Important Destination	Sub-core Destination
Leshan	Sub-core Destination	Sub-core Destination	Sub-core Destination	Important Destination
Nanchong	Important Destination	Sub-core Destination	Sub-core Destination	Important Destination
Suining	Important Destination	Sub-core Destination	Sub-core Destination	Important Destination
Meishan	Important Destination	Important Destination	Important Destination	Important Destination
Zigong	Important Destination	General Destination	Important Destination	Important Destination
Luzhou	Important Destination	General Destination	Important Destination	Important Destination
Ya’an	General Destination	General Destination	General Destination	Important Destination
Deyang	General Destination	Important Destination	Important Destination	General Destination
Guangyuan	General Destination	General Destination	General Destination	General Destination
Guang’an	General Destination	General Destination	General Destination	General Destination
Ganzi	General Destination	Edge Destination	General Destination	General Destination
Bazhong	General Destination	General Destination	General Destination	Edge Destination
Dazhou	Edge Destination	Edge Destination	General Destination	Edge Destination
Aba	General Destination	Edge Destination	General Destination	Edge Destination
Liangshan	General Destination	Edge Destination	General Destination	Edge Destination
Panzhihua	Edge Destination	Edge Destination	Edge Destination	Edge Destination

(1) Core Tourism Destinations: These are the pivotal nodes within the spatial network of tourism economics, serving as the central hubs for tourist flows. They rank highest in degree centrality, betweenness centrality, and closeness centrality within the region, possessing the greatest network power. They have the strongest capacity to attract external tourist flows and redistribute them to other areas within the province.
(2) Sub-Core Tourism Destinations: These act as intermediary nodes in the spatial network of tourism economics, exhibiting a relatively high degree centrality and betweenness centrality. They are crucial hubs connecting core nodes with other nodes, possessing a strong ability to attract external tourist flows. They receive tourist inflows from core nodes and disseminate them to other cities and states, functioning as significant transit stations for tourist flows.
(3) Important Tourism Destinations: These hold a moderate position within the spatial network of tourism economics, with a level of tourism economic development that is not as advanced as core nodes. They have a certain capacity to attract external tourist flows and serve as vital links between high and low-network status nodes.
(4) General Tourism Destinations: These occupy a lower position within the spatial network of tourism economics, with a weaker ability to attract external tourist flows and limited control over tourist flows to other cities and states. They rely on the tourist source output from core tourism destinations.
(5) Edge Tourism Destinations: These are the marginal nodes in the spatial network of tourism economics, with weak economic connections to other cities and prefectures. Their tourist flows are constrained by core nodes, and they cannot control tourist flows to other cities and states.

### 4.4. Analysis of influencing factors of spatial network structure of tourism economy

#### 4.4.1. Selection of influencing factor indicators.

The regional tourism economy has the characteristics of “distance decay” and “path dependence” (which represents administrative affiliation). The rigid constraints of spatial distance and administrative boundaries will affect the cooperation of the regional tourism economy [44]. The closer the spatial distance between tourism destinations, the more frequent the flow of tourists between them, and the relatively closer the economic links of tourism. The endowment of tourism resources is the core element in the formation of tourism products and is also a key factor in stimulating tourists’ travel motives, directly affecting the scale and level of development of the destination tourism industry. The quantity and quality of scenic spots owned by a tourist destination directly impact the development level and competitive ability of the tourism industry. The number of star-rated hotels is a direct reflection of the service reception capacity of a tourist destination. The level of economic development impacts the degree of spatial interaction and the development level of the economic network structure of the tourism economy [45]. The proportion of the tourism industry in the destination’s industrial structure will affect the government’s emphasis on the development of the tourism industry, further promoting the improvement of the regional tourism economy’s competitiveness by providing support in policies, funding, land, and other aspects of tourism industry development [46]. In addition, as a comprehensive and outward-looking industry, the level of a region’s openness to the outside world will also affect the inflow of tourism, thereby impacting the formation of tourism economic links and economic network structures.

Therefore, the selection of indicators such as geographical adjacency, shortest road distance, tourism resource endowment, tourism reception capacity, economic development level, degree of openness to the outside world, and industrial structure proportion, as the driving factors of the tourism economic spatial network structure in Sichuan Province. Among them, the economic development level is reflected by per capita GDP data, the shortest road distance is calculated by the shortest driving distance between cities and prefectures, tourism resource endowment is calculated by scoring and multiplying the number of 4A and 5A scenic spots in the region, tourism reception capacity is represented by the number of star-rated hotels, the degree of openness is reflected by the total import and export volume, and the industrial structure proportion is reflected by the proportion of tourism income to GDP. Difference matrices of the above indicators are constructed, and the QAP analysis method is used to analyze the explanatory power of the driving factors on the spatial network structure of the tourism economy. The QAP analysis method is based on the permutation of matrix relationship data and can eliminate false structural relationships through the construction of N × N matrices for correlation and regression analysis [19]. To avoid the influence of dimensional scale on the results, all difference matrices are standardized by range before QAP analysis.

#### 4.4.2. Analysis of results of influencing factors.

As shown in Table 9, the correlation between geographical adjacency and the shortest road distance with the intensity of tourism economic connections is the strongest, with P-values of 0.000, significant at the 0.01 level. The correlation coefficients are 0.446 and -0.472, respectively, and the regression coefficients are 0.290 and -0.329. There is a significant positive correlation between geographical proximity and the intensity of tourism economic connections, while the shortest road distance between cities and prefectures shows a significant negative correlation with the intensity of tourism economic connections. Cities and prefectures that are geographically adjacent are more likely to engage in cross-regional tourism cooperation, and due to their geographical proximity, the cost of tourism resource flow is also lower, resulting in closer tourism economic connections. Conversely, the greater the shortest road distance between cities and prefectures, the higher the time cost of spatial movement of tourist flows, and being farther from the regional core nodes is not conducive to receiving the radiation of tourist flows from the core area. Furthermore, regions nearby can share large-scale infrastructure (such as airports, conference centers, etc.) [47], which facilitates cross-regional tourism cooperation marketing, thereby forming spatial dependencies and micro-cooperation structures between regions, and influencing the development of regional tourism economic networks [48].

**Table 9:** Analysis of influencing factors of spatial network structure of tourism economy.

Independent variable	QAP related analysis		QAP regression analysis	
	Correlation coefficient	P value	Regression coefficient	P value
Geographical spatial proximity	0.446	0.000***	0.290	0.000***
Shortest road distance	-0.472	0.000***	-0.329	0.000***
Tourism resource endowment	0.110	0.010**	0.081	0.020**
Economic development level	0.116	0.002***	0.075	0.041**
Tourism reception capacity	0.120	0.002***	0.075	0.029**
Openness to the outside world	0.090	0.041	-0.001	0.483
Industrial structure proportion	-0.026	0.391	-0.042	0.154

Note: Adjusted R^2^ value decreased from 0.312 to 0.302, with a significance level of 0.000.

* , **, ***, respectively represent significance at the 0.1, 0.05, and 0.01 levels

The endowment of tourism resources and the capacity for tourism reception exhibit a significant positive correlation with tourism economic connections, with correlation coefficients of 0.110 and 0.120, and regression coefficients of 0.081 and 0.075, respectively. Tourism resources are the core attractions of a travel destination. The greater the quantity of high-quality tourism resources at a destination, the stronger the attraction to tourists, which enhances its ability to gather tourist flows and facilitates economic connections with other regions. Regions with a strong endowment of tourism resources gain cumulative advantages through external competition, which can create a trickle-down effect on regions with relatively scarce tourism resources, thereby elevating their status within the spatial network of tourism economics [48]. The hotel industry, as a core pillar of tourism, directly reflects regional tourism reception capacity through the scale and number of star-rated hotels. The development of the modern hotel industry has transcended its traditional focus on providing accommodation services, with an increasing number of hotels becoming new tourist attractions. High-quality tourism reception capacity meets the ever-evolving demands of tourists for enhanced travel experiences, exerting a pull on tourist flows [49].

The level of economic development positively influences the tourism economic connections among the 21 cities and prefectures in Sichuan Province. The construction of tourism infrastructure and service reception facilities is closely related to the level of regional economic development. Regions with higher economic development levels also have higher resident consumption levels, leading to greater expenditure on tourism. Areas with advanced economic development possess competitive advantages in tourism market scale, structure, and tourism policies and regulations, allowing them to occupy a more prominent position within the regional economic network [48]. The correlation between the degree of openness to the outside world, industrial structure proportion, and the intensity of tourism economic connections is not significant, with regression analysis probability P-values greater than 0.1, specifically 0.483 and 0.154. The degree of openness primarily considers Sichuan Province’s trade interactions with international markets. However, the overall scale of the inbound tourism market in Sichuan is relatively small, with a minor proportion of inbound tourist sources and foreign exchange earnings in total tourist numbers and total tourism revenue. The movement of inbound tourists among cities and prefectures within the province is minimal, thus having no impact on the intensity of tourism economic connections within the region. The proportion of tourism revenue to GDP reflects the economic contribution of tourism to the destination, which may be related to the fact that the tourism economic connections often occur between regions with similar tourism industry status [9].

## 5. Conclusion and discussion

### 5.1. Conclusion

This article focuses on Sichuan Province, China, as the study area and constructs a tourism economic spatial network matrix using the tourism gravity model. Employing social network analysis methods, it evaluates the power status of the tourism economic spatial network across 21 cities and prefectures in Sichuan Province, China. Furthermore, it elucidates the influencing factors of the tourism economic spatial network structure through the QAP analysis method. The main conclusions of the study are as follows:

(1) The volume of tourism economic connections is experiencing rapid growth, with the radiation capacity and spillover effects of core node areas continuously strengthening. Chengdu occupies a central position within the tourism economic connection network of Sichuan Province, exhibiting the strongest capacity to drive tourism in other regions. The spatial distribution of tourism economic connections in Sichuan Province is relatively concentrated, with Chengdu accounting for approximately 47% of the tourism economic connections with other areas in the overall region. Furthermore, there are significant disparities in tourism economic connection volumes among different cities and states, with the tourism economic connections in the Chengdu Plain Economic Zone being notably higher than in other regions. The impact of the COVID-19 pandemic has led to a significant reduction in the tourism economic connection volumes across the 21 cities and prefectures in Sichuan Province, resulting in a slight decline in the trend of spatial concentration of these connections.
(2) The overall density of the regional tourism economic spatial network is continuously increasing, with tourism economic connections evolving from a loose to a more cohesive trend. The radiating and driving effect of the main urban axis line of Chengdu-Deyang-Mianyang has been further strengthened, enhancing vertical tourism economic connections with Leshan and Meishan, while maintaining close horizontal interactions with Ziyang, Suining, Neijiang, Nanchong, Yibin, and Ya’an, thereby forming a spatial distribution pattern of tourism economic connections characterized by “one axis with multiple branches.” The COVID-19 pandemic has impacted the overall cohesion of Sichuan Province’s tourism economic spatial network, causing economic connections among regional members to become more dispersed. The spatial flow of tourist traffic is primarily centered around Chengdu, and cross-regional tourism cooperation has been restricted.
(3) There are variations in the centrality of tourism economic connection networks among cities and prefectures within the region, with different cities and prefectures occupying distinct positions and roles in the spatial network of tourism economics. Chengdu serves as the core node city of the tourism economic spatial network in Sichuan Province, possessing the highest level of network power and acting as the central hub for the aggregation and diffusion of tourist flows in the region. Mianyang and Yibin have shown significant increases in power within the tourism economic spatial network, while Neijiang, Nanchong, Suining, Leshan, Meishan, and Ziyang also exhibit a capacity to control tourist flows from other areas. In contrast, Bazhong, Dazhou, Aba, Ganzi, Liangshan, and Panzhihua hold lower positions of power in the tourism economic spatial network, exerting weaker influence on the tourism economic connections of other cities and prefectures.
(4) The formation and evolution of the spatial network structure of the tourism economy are influenced by factors such as geographical proximity, shortest road distance, level of economic development, endowment of tourism resources, and tourism reception capacity. Geographical proximity positively promotes tourism economic connections between regions, while the shortest road distance has a negative impact on these connections. Regions with superior tourism resource endowments possess a strong ability to attract tourist flows, making it easier for them to establish tourism economic links with other areas. The level of economic development and tourism reception capacity are key driving factors in the formation of regional tourism economic spatial networks. The impact of the level of openness to the outside world and the proportion of industrial structure on the spatial network structure of the tourism economy is not significant.

### 5.2. Discussion

(1) The rapid and efficient tourism transportation network can reduce the time cost of tourist transport, promote the economic exchange capability of tourism resource elements, and promote the role of core city tourism economy radiation. The construction of the high-speed rail network in Sichuan Province should be accelerated to promote the formation of a high-speed rail network pattern. Improve the construction of the high-speed rail networks between Chengdu and various cities and prefectures, and harness the spillover effects of Chengdu’s tourism resources, thereby shifting the development of Chengdu’s tourism economy from a polarization effect to a diffusion effect. Develop the vertical secondary transportation axes connecting Nanchong, Suining, Neijiang, Zigong, and Yibin (Luzhou) to strengthen the tourism economic connections between the northern and southern regions of Sichuan Province. Establish Neijiang and Suining as tourism distribution centers that facilitate the east-west and north-south transportation axes across Sichuan Province.
(2) The adjacency of geographical spaces plays a significant role in the coordinated development of regional tourism. Adjacent cities and prefectures are more likely to engage in cross-regional tourism cooperation, and due to their geographical proximity, the cost of tourism resource element flow is also relatively low. Therefore, it is necessary to leverage the advantage of geographical proximity between cities and prefectures to carry out regional cooperation in tourism infrastructure construction, tourism resource development and planning, tourism route design and optimization, as well as tourism marketing and promotion. Cities and prefectures located on the geographical edge of Sichuan Province should actively strengthen their spatial ties with neighboring cities and prefectures in the tourism economy, serving as the host for the tourism radiation of neighboring cities or as a conduit for the regional center city through neighboring cities.
(3) Exploring the cultural connotations of tourism resources and cultivating new types of tourism products can enhance the attractiveness of tourist destinations. Each city and prefecture should fully tap into the value of tourism resources, aligning with market demands and resource advantages to develop tourism products and activities with regional characteristics. Strengthening the development and cooperation of large-scale tourism routes between neighboring cities and prefectures is essential, in planning to form attractive cross-regional tourism routes. Introducing renowned domestic and international tourism groups, as well as cultural performances, theme parks, and other brand enterprises to operate chains in various cities and prefectures, can optimize the governance capabilities and development environment of tourism market entities. Improve the construction of supporting tourism service facilities to elevate the reception service standards of tourist destinations.
(4) It is necessary to give full play to the role of Chengdu as a core tourist destination in gathering and radiating tourism, and to strengthen the Greater Chengdu Urban Tourism Circle by taking a point-to-surface approach. Enhance the tourism distribution and transit capabilities of sub-core tourist destinations such as Leshan, Nanchong, and Suining. Improve the tourism service supply capacity of important tourist destinations like Neijiang, Mianyang, Yibin, and Ziyang to increase the length of stay for visitors at the destinations. Accelerate the construction of transportation infrastructure for general and edge tourist destinations to improve external accessibility. Intensify tourism marketing efforts to boost the attractiveness of destinations.

### 5.3. Limitations and prospects

This paper employs social network analysis methods to study network structures based on relational data, thereby enriching the research perspective on the spatial structure of the tourism economy. Compared to traditional studies of tourism spatial structures that use attribute variables, employing relational variables allows for a more accurate depiction of the overall network characteristics of regional tourism economies Network structure theory and methods represent a novel quantitative approach to studying tourism spatial structures, particularly by analyzing relevant indicators to determine the position and role of tourist destinations within the network, thus providing a clearer understanding of the spatial network structure of tourist destinations [50]. However, the article has certain limitations: (1) The tourism economic gravity model is significantly influenced by transportation distance, which may overlook the impact of other factors. Future research should consider both transportation spatial distance and time distance when selecting distance indicators. (2) The social network analysis method requires binarization (0-1) of the relational matrix, which may lead to some data loss. When processing data for dichotomization, it is crucial to select the cut-off values more scientifically and rationally. (3) Tourism flow is a complex spatial system that includes multiple tourism elements such as tourist flow, information flow, and transportation flow. This paper analyzes the spatial structure characteristics of the tourism economy solely from the perspective of tourism economic connections. Future research could conduct comparative studies of tourism spatial network structures from a multi-flow perspective.

## Supporting information

S1 File Tourism economic connectivity in Sichuan Province from 2015 to 2022. (ZIP)
